# Cerebrospinal Fluid Total Tau Protein Correlates With Longitudinal, Progressing Cognitive Dysfunction in Anti-Neural Autoantibody-Associated Dementia and Alzheimer’s Dementia: A Case–Control Study

**DOI:** 10.3389/fimmu.2022.837376

**Published:** 2022-03-03

**Authors:** Niels Hansen, Aaron Levin Juhl, Insa Maria Grenzer, Sina Hirschel, Bianca Teegen, Dirk Fitzner, Claudia Bartels, Charles Timäus, Jens Wiltfang, Berend Malchow

**Affiliations:** ^1^ Department of Psychiatry and Psychotherapy, University Medical Center Göttingen, Göttingen, Germany; ^2^ Euroimmun Reference Laboratory, Lübeck, Germany; ^3^ Department of Neurology, University of Göttingen, Göttingen, Germany; ^4^ German Center for Neurodegenerative Diseases (DZNE), Göttingen, Germany; ^5^ Neurosciences and Signaling Group, Institute of Biomedicine (iBiMED), Department of Medical Sciences, University of Aveiro, Aveiro, Portugal

**Keywords:** autoimmunity, dementia, neural autoantibody, neurodegeneration, cognitive decline

## Abstract

**Background:**

Neural autoantibody-associated dementia (NABD) is an increasing phenomenon in memory clinics with a high impact on later therapy. Biomarkers are lacking that differentiate this type of dementia from neurodegenerative dementia such as Alzheimer’s dementia (AD). Our aim is to analyze neurodegeneration markers and their relationship to progressing cognitive dysfunction in NABD and AD to test for tools differentiating these two forms of dementia prior to neural autoantibody testing.

**Methods:**

In our retrospective, observational study, we investigated 14 patients with dementia and serum and/or cerebrospinal fluid (CSF) neural autoantibodies as well as 14 patients with AD by relying on recent CSF and clinical criteria for AD. Patient files were checked for psychopathology, neuropsychological test performance, autoimmune indicators, CSF, and MRI results.

**Results:**

Our patient groups did not differ in their psychopathology, autoimmune indicators, or MRI profile. The progression of cognitive dysfunction [as measured by the difference in Mini-Mental State Examination (MMSE) scores since disease onset, and the yearly progression rate (MMSE loss/per year)] did not vary significantly between groups. Total tau protein was significantly higher in AD patients than NABD patients revealing no signs of Alzheimer’s disease pathology in their CSF (p < 0.05). Total tau protein levels in CSF correlated with cognitive decline since disease onset (r = 0.38, p < 0.05) and yearly progression rates (r = 0.56, p < 0.005) in all patients.

**Discussion:**

Our results suggest that the progression of cognitive dysfunction as defined by MMSE does not seem to be an appropriate biomarker for distinguishing NABD from AD. However, the total tau protein level in CSF might be a relevant molecular biomarker that can indicate disease pathology and/or progression in both known AD and NABD, which is often accompanied by axonal degeneration. Total tau protein may be an additional diagnostic tool with which to differentiate anti-neural-associated dementia from AD if further research confirms these proof-of-concept findings in larger patient cohorts.

## Introduction

Neural autoantibody-associated dementia (NABD) (1, 2) is a disease entity whose incidence is rising through the identification of novel neural autoantibodies in relationship to a clinical phenotype characterized by initial cognitive impairment. Recent progress has been made in classifying NABD (2–4). If certain criteria are fulfilled such as inflammation in cerebrospinal fluid (CSF), an imaging pattern in MRI atypical for neurodegenerative disease, and a good response to immunotherapy, autoimmune dementia should be assumed in agreement with the Flanagan et al. (4) criteria. Various neural autoantibodies have been described to be associated with dementia. Neural autoantibodies against the cell surface such as *N*-methyl-d-aspartate receptor (NMDAR), gamma aminobutyric acid B receptor (GABABR), α-amino-3-hydroxy-5-methyl-4-isoxazolepropionic acid receptor (AMPAR), leucine-rich glioma inactivated protein 1 (LGI1), and dipeptidyl-peptidase-protein-like 6 (DPPX) are often detected in patients suffering from worsening cognitive impairment (2, 3). However, there have been no investigations addressing the progression of cognitive dysfunction in autoantibody-associated dementia compared to Alzheimer’s dementia (AD). Recent individual case reports (5–7) and reviews (1, 3) have postulated that rapidly progressing cognitive dysfunction associated with neural autoantibodies are a typical clinical manifestation of NABD. We thus conducted a pilot trial relying on retrospective data to identify whether progressing cognitive dysfunction is more prominent in NABD than in AD. Our second point of inquiry concerned the axonal neurodegeneration detected in some patients with neural antibody-associated dementia (2, 8–11) to see if tau pathology is associated with rapidly worsening cognitive impairment in both dementia types. We wondered 1) whether the degree of axonal neurodegeneration differs in NABD from that in AD and 2) if the axonal degeneration correlates with the longitudinal time course of cognitive dysfunction. To answer these questions, we analyzed retrospective data including neurodegenerative markers such as tau protein and phosphorylated tau protein 181 (ptau181) and correlated those with cognitive decline as determined *via* Mini-Mental State Examination (MMSE) scores.

## Methods

In this retrospective, observational cohort study, we enrolled 14 patients with mild-to-moderate dementia and presenting neural serum or CSF antibodies (NABD). We screened in- and outpatients with dementia in our Department of Psychiatry and Psychotherapy, University Medical Center Göttingen. Dementia was diagnosed according to its definition in the *Diagnostic and Statistical Manual of Mental Disorders*, fifth edition (DSMV) (12), suggesting a cognitive dysfunction entailing disturbed higher cortical function in conjunction with impaired daily living activities. We selected an age- and gender-matched group of molecular CSF biomarker-based AD patients with dementia from the biomaterial bank of the Department of Psychiatry and Psychotherapy relying on the Jack et al. criteria (13). The AD group was screened 1) *via* a CSF profile suggesting AD according to Jack et al. (13) and 2) a typical AD phenotype as recently described by Dubois (14). We considered a reduced β-amyloid 42/40 ratio (Aβ42/40) and elevated ptau181 as being typical of Alzheimer’s disease pathology and concurring with the Jack et al. (13) criteria. To assess neurodegeneration markers, we relied on these normative values: a level is considered as non-pathological level if 1) tau protein <450 pg/ml, 2) ptau181 <61 pg/ml, 3) β-amyloid 42 (Aβ42) >450 pg/ml, and 4) ratio Aβ42/Aβ40 ×10 >0.5. CSF total tau and ptau181 levels were manually quantified utilizing commercial ELISA from Fujirebio (Tokyo, Japan) [INNOTEST hTAU-Ag; INNOTEST PHOSPHO TAU (181P)]. CSF Biomarker Aβ42 was manually assessed using the commercially available INNOTEST^®^ β-AMYLOID (1-42) ELISA kit (Fujirebio). CSF Aβ40 was manually quantified *via* commercially available ELISA from IBL [AMYLOID BETA (1–40)]. The normative values for ptau181, tau, Aβ42, Aβ40, and ratio Aβ42/Aβ40 we referred to rely on the (unpublished, in-house) normative laboratory values from the Neurochemistry Laboratory, Neurology Department, University Medical Center Göttingen. Determining the Aβ42/40 ratio requires expertise but has proven to be superior in diagnosing AD to Aβ42 alone (15, 16). We relied on the McKeith criteria (17) to classify dementia with Lewy bodies (DLB). The Rascovsky et al. criteria (18) were used to determine the behavioral variant of frontotemporal dementia (bvFTD), the Gorno-Tempini criteria (19) were utilized to classify primary progressive aphasia, and the Flanagan et al. (4) criteria served to classify autoimmune dementia (4). We assessed weak and strong autoimmune indicators according to published guidelines (20). Specific neural autoantibodies were investigated using BIOCHIP mosaics consisting of brain tissue and recombinant cells. BIOCHIP mosaics contain human embryonic kidney cells transfected with neural antigens to test serum or CSF biological probes. We ran standard immunofluorescence tests to test autoantibodies against intracellular antigens such as amphiphysin, ANNA-3, CV2, glutamic acid decarboxylase (GAD65), TR, Ma1/Ma2, Ri, SOX1, Yo, Zic4, and HuD. We also carried out immunofluorescence tests for autoantibodies against cell-surface antigens such as NMDAR, AMPAR1/2, GABABR, LGI1, DPPX, contactin-associated protein 2 (CASPR2), aquaporin 4, and flotillin 1/2. For glycine receptors, gamma aminobutyric acid receptor (GABAAR), recoverin, potassium voltage-gated channel subfamily A member 2 (KCNA2), and flotillin 1/2, we ran homemade immunofluorescence tests from the Euroimmun laboratory. Cell-based assays were also done for all autoantibodies except ANNA3- and anti-myelin. The Euroimmun laboratory in Lübeck did all the neural autoantibody testing, which were measured semiquantitatively differentiating between low-, medium-, and high-intensity levels in their respective biological probes. CSF probes were analyzed in the Neurochemistry Laboratory of the Neurology Department, University Medical Center Göttingen. Neuroimaging data were retrieved from patient files consisting of 1.5-Tesla MRI done in the Department of Neuroradiology in the University Medical Center Göttingen or off-site at different neuroradiological medical centers. We relied on the AMPD System [*Manual for Assessment and Documentation of Psychopathology in Psychiatry*, 9th edition, (21)] to assess psychopathology with the following scoring system: score “1” means the symptom is present, whereas score “0” refers to the non-presence of the symptom. The geriatric depression scale was employed to assess depressive symptoms in patients with dementia. We considered a value above 5 as mild and above 10 as severe depression. This retrospective, observational study concurs with the latest Declaration of Helsinki and was approved by our Ethics Committee.

### Assessing the Progression of Cognitive Dysfunction

Cognitive function was assessed by the CERAD (Consortium to Establish a Registry for Alzheimer’s Disease) including the MMSE. To assess global cognitive decline, we opted not to use data from the complete CERAD testing as that data were available on 21 patients, whereas our MMSE data covered more patients (n = 26). Note that as we have so few and inconsistent CERAD testing follow-ups from our groups, we relied on CERAD testing only to describe cognitive functions cross-sectionally in patient groups. AD progression was quantified by worsening cognitive dysfunction noted in the patients’ MMSE scores. An average drop in the MMSE ≥ 3 points per year was considered as rapidly progressing cognitive dysfunction according to an approximately average cognitive loss in AD about 3 MMSE items per year (22). The onset age of disease was defined as the last observation of normal cognitive function with an MMSE score of 30. The patient’s first MMSE score at their initial, baseline clinical presentation was subtracted from the 30 MMSE value at the onset of cognitive dysfunction. The loss of cognitive dysfunction per year was calculated as the difference in MMSE scores at the patient’s current disease stage and at disease onset (MMSE = 30)—that amount was then divided by the years of disease duration. If a score dropped by 3 MMSE items per year, we assumed a rapidly progressing cognitive dysfunction, which was given “1” as their score. However, if the patient’s MMSE score dropped by fewer than 3 items per year, we assumed no cognitive dysfunction progression, which was allocated a “0.” We then calculated the percentage of patients in each group (NABD vs. AD) demonstrating a rapid progression. The MMSE scores over time of those patients whose MMSE scores improved were added together, and their percentage was then calculated.

### Statistical Analysis

Normally distributed data were compared between groups by Student’s *t*-test, whereas non-normally distributed data were analyzed by the non-parametric Mann–Whitney U-test including a Bonferroni correction. Frequencies for dichotomy scores (1 = item is present, 0 = item not present) (psychopathology, MRI scores, autoimmune indicators, and immune challenge) were calculated by Fisher’s exact test. Correlations between cognitive dysfunction and levels of CSF markers of neurodegeneration were drawn by Spearman’s rho correlation test for non-parametric data. Statistical relevance was declared if the p-level was below 0.05.

## Results

### Classification of Patient Groups

**Figure 1:**
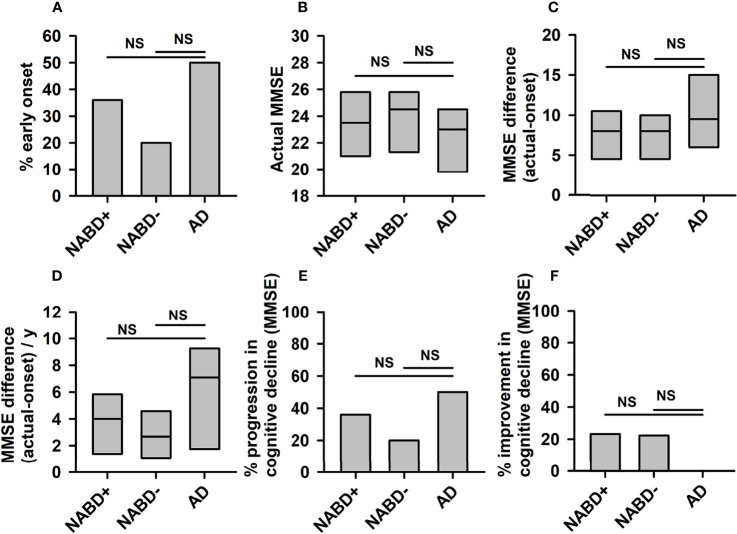
Progression of cognitive dysfunction in neural autoantibody-associated dementia and Alzheimer’s dementia. No significant differences between NABD+ or NABD− and AD were detected regarding the percentual proportion of patients with an early-onset **(A)**, current MMSE **(B)**, MMSE difference between onset and current stage without **(C)** and with division per years of disease duration **(D)**, the percentual proportion of patients with rapid progression per year **(E)**, and the percentual proportion of patients with an improvement over time **(F)**. AD, Alzheimer’s dementia; CSF, cerebrospinal fluid; MMSE, Mini-Mental State Examination; NABD+, neural autoantibody-associated dementia including patients with CSF-based Alzheimer’s disease pathology; NABD−, anti-neural autoantibody associated dementia without cerebrospinal fluid-based Alzheimer’s disease pathology; NS, non-significant.

Our neural autoantibody group consisted of 14 patients with dementia and autoantibodies in their serum or CSF (serum: n = 3 recoverin, n = 2 KCNA2, n = 1 GFAP, n = IgLON5, n = 1 Zic4, n = 1 glycine, n = 1 CASPR2, n = 1 Ma2, n = 1 SOX1, n = 1 titin, n = 1 flotillin 1/2, n = 1 Yo antibodies, n = 1 neuropil antibodies, and CSF n = 1 neuropil antibodies). The time between the dementia diagnosis and neural autoantibody assessments did not differ between groups (NABD +0.39 ± 0.46 years versus AD −0.43 ± 1.1 years). Moreover, the time between disease onset as cognitive impairment and determining neural autoantibodies did not differ between groups (NABD 2.7 ± 2.1 years versus AD 1.8 ± 1.8 years). The percentage of patients suffering an early-onset did not differ between groups (**Figure 1A**). However, although neural autoantibodies were only assessed in 7/14 (50%) of the AD patient group, no neural autoantibodies were detected in any of them. We thus detected no neural autoantibodies in our AD patient group, but all our NABD patients did present them (neural autoantibodies as a group criterion for NABD). The clinical phenotype of NABD patients varied from autoimmune dementia (n = 4), atypical dementia (n = 6), DLB (n = 1) to frontotemporal lobar degeneration (n = 3). We observed a CSF profile but inconclusive clinical features concurring with AD in four NABD patients who presented a reduced Aβ42/40 ratio and elevated ptau181 in CSF. Fourteen biomarker-based patients and the clinical AD phenotype served as our disease control group. Age, age of disease onset, years of disease duration, sex, psychopathology, type of autoimmune indicators, CSF cells and proteins, blood–brain barrier disturbance score, oligoclonal bands score, and MRI data did not differ between groups (**Table 1**). We found none of these psychopathological conditions in any patient in our groups: consciousness disturbances, worries and compulsions, ego disturbances, and self-harm. Furthermore, we detected no autoimmune indicators such as autonomic disturbances, central hypoventilation, decreased level of consciousness, epileptic seizures, faciobrachial dystonic seizures, hyponatremia, infectious prodrome, new-onset headache, adverse response to psychopharmacologic drugs, other autoimmune disorder or the presence of neuroleptic malignant syndrome, early resistance to therapy, or fluctuating psychopathology in any patient of our patient groups. If we exclude the four patients with AD pathology in CSF from the NABD group, there were no differences between the NABD without CSF AD pathology (NABD−) and the purely AD patients in age, age of onset, years of disease duration, sex, psychopathology, type of autoimmune indicators, CSF cells and proteins, blood–brain barrier disturbance score, oligoclonal bands score, and MRI data (**Table 1**).

**Table 1:** Clinical characterization of patient groups.

Parameter	NABD		AD
	1. NABD+	2. NABD−	
***Demographic parameter***			
Sex (female)	7/14 (50%)	6/10 (60%)	8/14 (57%)
Age, years	n = 14, 72.6 ± 2.7	n = 10, 69.7 ± 3	n = 14, 72 ± 3
Age of onset, years	n = 14, 68 ± 3	n = 10, 65.5 ± 3.4	n = 14, 64 ± 2.9
Early onset n	5/14 (35.7%)	2/10 (20%)	7/14 (50%)
Age at first neuropsychology testing, years	n = 14, 71.3 ± 2.9	n = 10, 68.1 ± 3.2	n = 14, 68 ± 3.1
Rapid progression of cognitive impairment	7/14 (50%)	4/10 (40%)	8/14 (57%)
Improvement of cognitive impairment	3/14 (22%)	2/10 (20%)	0/14 (0%)
***Psychopathology***			
Disturbances of orientation (0–4)	n = 14, 1.1 ± 0.9	n = 10, 1.1 ± 1.0	n = 14, 0.6 ± 1.1
Disturbances of attention and memory (0–6)	n = 14, 3.3 ± 0.6	n = 10, 3.3 ± 0.7	n = 14, 3.1 ± 0.5
Formal thought disorder (0–12)	n = 14, 0.6 ± 0.7	n = 10, 0.7 ± 0.8	n = 14, 0.7 ± 0.7
Delusions (0–6)	n = 14, 0.01 ± 0.04	n = 10, 0.01 ± 0.04	n = 14, 0.1 ± 0.3
Disorders of perception (0–6)	n = 14, 0	n = 10, 0	n = 14, 0.01 ± 0.04
Disturbances of affect (0–21)	n = 14, 0.71 ± 0.9	n = 10, 0.9 ± 1.0	n = 14, 1.4 ± 1.5
Disorders of drive, psychomotor activity (0–9)	n = 14, 0.5 ± 0.7	n = 10, 0.5 ± 0.7	n = 14, 0.4 ± 0.5
Social withdrawal	2/14 (14%)	2/10 (17%)	1/14 (7%)
Aggressiveness	2/14 (14%)	1/10 (10%)	0/14 (0%)
Suicidal behavior	1/14 (7%)	1/10 (10%)	2/14 (14%)
***Strong indicators for autoimmunity***			
Aphasia, mutism, dysarthria	2/14 (14%)	2/10 (20%)	0/14 (0%)
Focal neurological deficit	3/14 (21%)	1/10 (10%)	1/14 (7%)
Movement disorder	1/14 (7%)	1/10 (10%)	1/14 (7%)
Optic hallucinations	0/14 (0%)	0/10 (0%)	1/14 (7%)
Paresthesia	1/14 (7%)	0/10 (0%)	0/14 (0%)
Presence of a tumor	1/14 (7%)	0/10 (0%)	0/14 (0%)
Severe cognitive dysfunction	14/14 (100%)	10/10 (100%)	14/14 (100%)
*Sum score (0–18)*	*n = 14, 1.6 ± 0.9*	*n = 10, 1.4 ± 0.5*	*n = 14, 1.1 ± 0.5*
***Weak indicators for autoimmunity***			
Confusion	1/14 (7%)	1/10 (10%)	0/14 (0%)
Dynamic course	1/14 (7%)	1/10 (10%)	0/14 (0%)
*Sum score (0–4)*	*n = 14, 0.1 ± 0.5*	*n = 10, 0.2 ± 0.6*	*n = 14, 0 ± 0*
***CSF***			
Cell count (<5 µg/L)	n = 13, 1.0 (0.0/1.5)	n = 9, 0.0 (0.0/1.5)	n = 14, 1.0 (0.0/1.25)
Albumin, mg/L	n = 13, 287 ± 89	n = 9, 267 ± 102	n = 13, 264 ± 79
IgG, mg/L	n = 13, 31.0 (22.9/46.6)	n = 9, 33.8 (17.5/54.3)	n = 13, 29.3 (20.8/30.5)
IgA, mg/L	n = 13, 3.8 (2.1/6.5)	n = 9, 3.8 (2.1/5.5)	n = 13, 3.9 (1.8/5.9)
IgM, mg/L	n = 13, 0.5 (0.3/0.9)	n = 9, 0.4 (0.2/0.8)	n = 13, 0.29 (0.1/0.4)
Tau protein (<450 pg/ml)	n = 13, 528 ± 233	n = 9, 458.4 ± 198*	n = 14, 820 ± 309
P tau protein 181 (<61 pg/ml)	n = 13, 94 ± 49	n = 9, 79.2 ± 31	n = 14, 107 ± 28
Aβ42 (>450 pg/ml)	n = 13, 925 ± 496	n = 9, 1075 ± 530	n = 14, 563 ± 126
Aβ40	n = 13, 12206 ± 3028	n = 9, 11662 ± 2520	n = 14, 14343 ± 3108
Ratio Aβ42/40 ×10 (>0.5)	n = 13, 0.55 (0.5/1.3)*	n = 9, 0.76 (0.6/1.4)*	n = 14, 0.39 (0.4/0.4)*
***Brain MRI***			
Generalized atrophy	6/12 (50%)	3/8 (38%)	1/8 (12.5%)
Focal atrophy	7/13 (56%)	5/8 (62.5%)	5/8 (62.5%)
Vascular lesions	7/12 (58%)	5/8 (62.5%)	5/8 (62.5%)
***Neuropsychological test scores***			
MMSE (sum score)	n = 12, 23.5 (21/25.8)	n = 8, 24.5 (21.3/25.8)	n = 14, 23 (19.8/24.5)
CERAD Boston naming test (z-score)	n = 12, −1.4 ± 2	n = 8, −1.09 ± 1.71	n = 12, −1 ± 1.3
CERAD semantic fluency (z-score)	n = 12, −1.6 ± 1.3	n = 8, −1.6 ± 1.07	n = 12, −1.9 ± 1.4
CERAD phonemic fluency (z-score)	n = 11, −1.4 ± 1.2	n = 8, −1.8 ± 1.24	n = 12, −1.14 ± 1.4
CERAD list learning (trials 1-3) (z-score)	n = 12, −2.8 ± 1.6	n = 8, −2, 8 ± 0.82	n = 12, −2.8 ± 1.2
CERAD list recall (savings) (z-score)	n = 12, −2.1 ± 2.1	n = 8, −2.4 ± 0.99	n = 12, −2.8 ± 1.9
CERAD recognition/discriminability (z-score)	n = 11, −1.1 ± 1.5	n = 8, −1.39 ± 1.09	n = 11, −1.8 ± 1.3
CERAD figure recall (savings) (z-score)	n = 11, −1.8 ± 1.3	n = 8, −1.71 ± 1.9	n = 11, −1.3 ± 1.7
CERAD figure copy (z-score)	n = 12, −0.5 ± 1.5	n = 8, −0.43 ± 1.67	n = 11, −2.2 ± 1.8
TMT part A (z-score)	n = 11, −0.75 ± 1.1	n = 8, −0.83 ± 1.05	n = 11, −1.6 ± 1.2

The values are depicted as mean ± SD. For laboratory data, normal ranges are shown in brackets. For neuropsychological testing, z-values as normative data are shown unless otherwise indicated. z-Values < −1 indicate performance below the normal range, and z-values ≥ −1 exhibit performance within the normal range.

AD, Alzheimer’s disease dementia; CERAD, The Consortium to Establish a Registry for Alzheimer’s Disease; CSF, cerebrospinal fluid; GDS, Geriatric Depression Scale; IgA, immunoglobulin A; IgG, immunoglobulin G; IgM, immunoglobulin M; MMSE, Mini-Mental State Examination; NABD, Neural autoantibody-associated dementia; NABD+, anti-neural autoantibody associated dementia including patients with Alzheimer pathology based on the cerebrospinal fluid; NABD−, anti-neural autoantibody associated dementia without patients with Alzheimer pathology based on the cerebrospinal fluid; P tau protein 181, phosphorylated tau protein 181; TMT, Trail Making Test. *p < 0.05.

### Cognitive Dysfunction and Progression in Both Groups

AD and NABD+ (NABD patients including 4 patients with CSF indices of Alzheimer’s disease pathology) demonstrating or NABD− patients’ first, baseline clinical MMSE scores did not differ (NABD, 22.2 ± 1.2 MMSE; NABD−, 23.8 ± 2.1 MMSE; AD, 22.4 ± 1.1 MMSE, n.s.; **Figure 1B**). Calculating the duration of cognitive decline in years since the first manifestation of cognitive dysfunction, there was no mean difference in MMSE scores of NABD+ or NABD− versus AD (NABD+, −8.5 ± 1.4; NABD, −7.7 ± 3.5; AD, −10.9 ± 0.25, n.s.; **Figure 1C**). In addition, the yearly cognitive decline expressed as MMSE difference/year did not differ between NABD+ or NABD− and AD patients (**Figure 1D**). The number of patients with NABD+ or NABD− and rapidly progressing cognitive dysfunction did not differ from that with AD (54% in NABD+ vs. 57% in AD, and 40% in NABD− vs. AD; **Figure 1E**). Furthermore, we observed improved cognitive function in 23% of patients with NABD+ and in 20% of those with NABD−, but none in those with AD within the disease time course (**Figure 1F**). However, the percentages of patients with NABD+ or NABD− whose MMSE scores rose within the disease time course did not differ significantly from AD patients. These results were confirmed in NABD+ or NABD− patients, unlike in AD patients.

### Markers of Neurodegeneration and Amyloidopathy Between Groups

**Figure 2:**
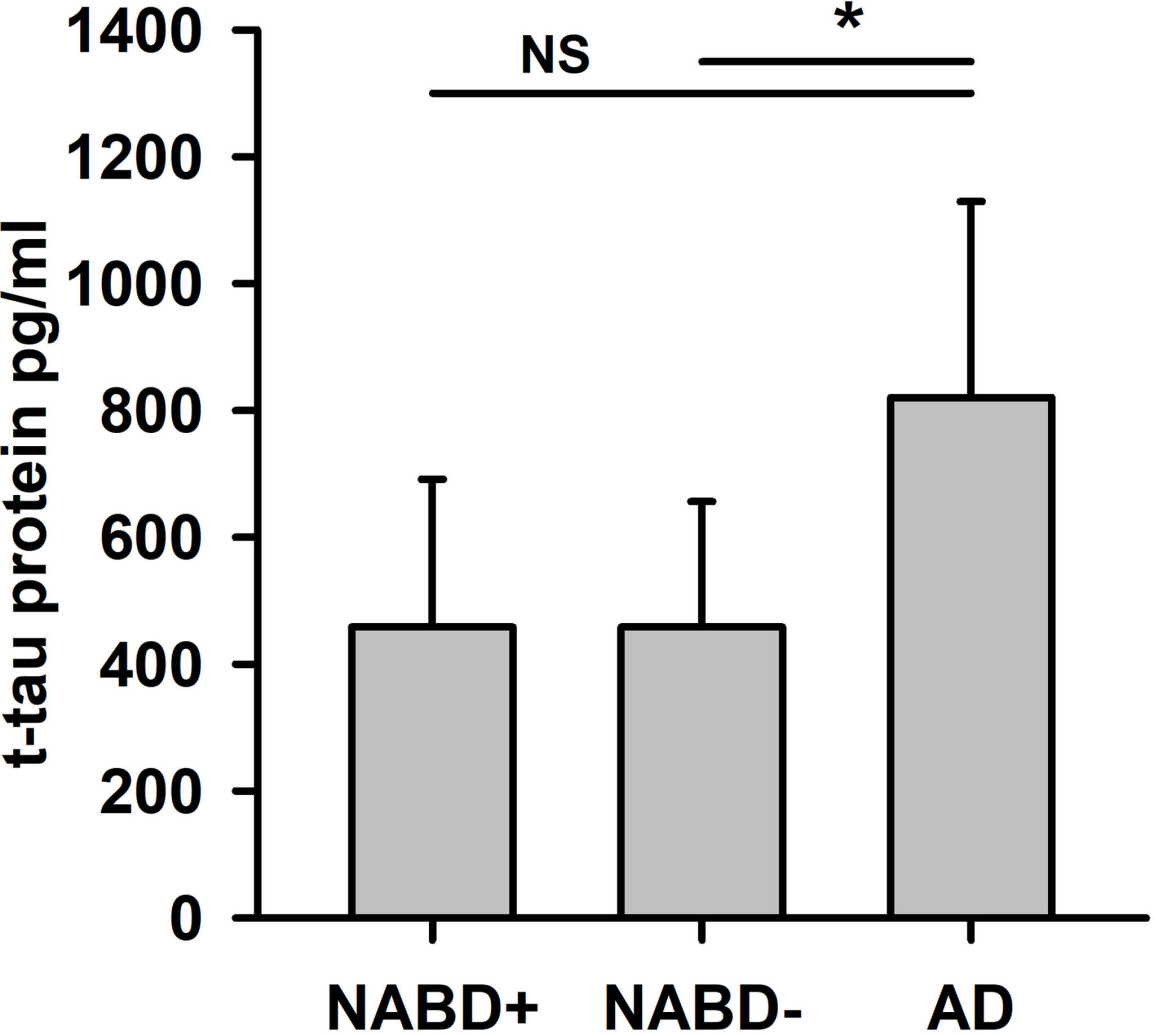
Total tau protein is higher in AD compared to patients with neural-associated dementia. Total tau protein (t-tau protein) in cerebrospinal fluid (CSF) is significantly different in NABD− versus AD, but not in NABD+ versus AD patients. AD, Alzheimer’s dementia; NABD+, neural autoantibody-associated dementia including patients with CSF-based Alzheimer’s CSF-based Alzheimer’s disease pathology; NABD−, anti-neural autoantibody associated dementia without CSF-based Alzheimer’s disease pathology; CSF, cerebrospinal fluid. *p < 0.005. NS, non-significant.

We detected a reduced Aβ42/40 ratio in the AD group, but not in the NABD group (**Table 1**, Mann–Whitney U test, p < 0.005). The reduced Aβ42/40 in the AD group is not surprising, as it was one of the AD group’s inclusion criteria. In the NABD group, both tau and phosphorylated tau proteins were elevated in 71.4% (tau protein in 35.7% and ptau181 in 71.4%) and in 100% of those with AD (tau protein in 86% and ptau181 in 100%). However, the tau protein and ptau181 values in the AD and NABD groups did not differ (**Table 1**), indicating that axonal neurodegeneration might also occur in NABD. However, after excluding the patients with AD pathology in the CSF (NABD−), total tau protein levels (but not phosphorylated tau protein) were significantly lower in the NABD than in the AD group (NABD total tau protein 458.4 ± 198 pg/ml versus total tau protein in AD 820 ± 309 pg/ml, p < 0.005, **Table 1** and **Figure 2**).

### Correlation Between Neurodegeneration and Amyloidopathy Markers in Conjunction With Long-Term and Yearly Cognitive Decline

**Figure 3:**
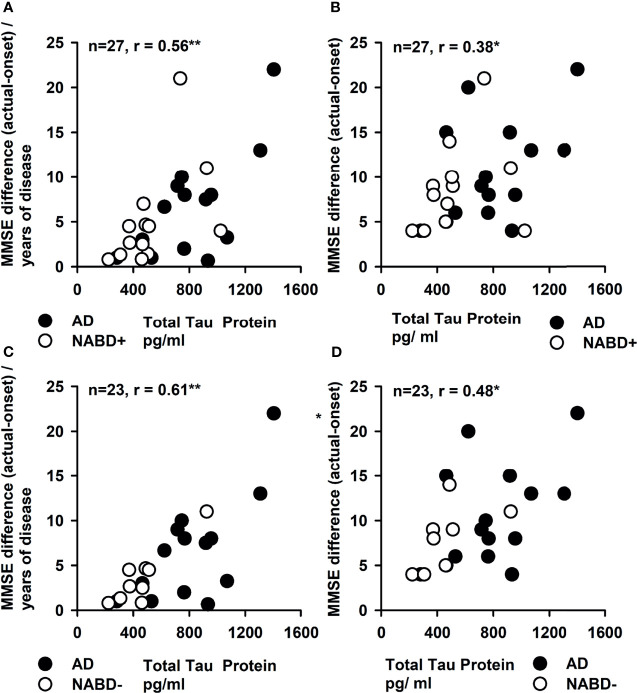
Correlation of cerebrospinal fluid total tau protein and yearly and total progression of cognitive dysfunction. The yearly progression of cognitive dysfunction depicted as mean Mini-Mental State Examination (MMSE) difference between the MMSE at disease onset and current MMSE (MMSE difference) divided per year was strongly correlated with the total tau protein in CSF in **(A, C)**. **(B, D)** The total tau protein in cerebrospinal fluid (CSF) is correlated with the MMSE difference. In all figures, both groups [NABD+ and AD **(A, B)** as well as NABD− and AD **(C, D)**] are correlated as one group between total tau protein and yearly and total progression of cognitive decline. AD, Alzheimer’s dementia; NABD+, neural autoantibody-associated dementia including patients with CSF-based Alzheimer’s disease pathology; NABD−, anti-neural autoantibody-associated dementia without cerebrospinal fluid-based Alzheimer’s disease pathology. *p < 0.05, **p < 0.005.

Total tau protein values correlated with the yearly (r = 0.56, p < 0.005; **Figure 3A**) and long-term cognitive decline in both groups (MMSE difference in follow-up compared to MMSE baseline) when investigated as a single group (r = 0.38, p < 0.05; **Figure 3B**) (AD and NABD+: n = 27), but not when subgroups (AD or NABD+) were investigated separately. Furthermore, total tau protein correlated positively with yearly (r = 0.61, p < 0.005, **Figure 3C**) and long-term cognitive loss (r = 0.48, p < 0.05; **Figure 3D**) in NABD− group in conjunction with the AD group. ptau181, Aβ42, and the Aβ42/40 ratio did not correlate with yearly decline, long-term decline, or the patients’ current degree of cognitive dysfunction. Moreover, the latest MMSE scores did not correlate with total tau protein in CSF in patients with NABD and AD.

## Discussion

NABD is a disease entity affecting a very heterogeneous group of patients with different neural autoantibodies that often underlies distinct pathomechanisms, which in turn depend on specific neural cell-surface antibodies. The data from our outpatient memory clinic fail to confirm the literature-based hypothesis (3) that patients with NABD experience faster progression than others (i.e., those with AD). Furthermore, our data on longitudinal cognitive decline in patients with NABD suggest a progression that resembles that in AD. Although the cognitive decline in NABD patients progresses faster than the usual progression in AD, that is, losing three MMSE points per year, it does not differ from age- and sex-matched AD patients (as a disease control group) with a similar age of onset. The immune dysregulation and activation caused by autoantibodies induce a similar deterioration in brain functions originating from different autoantibodies on channels, as occurs in patients with neurodegenerative dementia such as AD. We observed axonal neurodegeneration in 71.4% as evidenced by elevated total tau protein and ptau181 of our NABD patients—substantial neurodegeneration that might suffice to drive a rapid cognitive decline in most NABD patients. Autoantibody-associated cognitive decline is reported to begin with a subacute cognitive impairment onset that soon develops into rapid cognitive decline (2, 3, 5–7). No large cohort studies exist on long-term cognitive decline in NABD. Our results therefore contradict the latest findings on NABD concerning the progression of long-term cognitive decline, namely, that it is not faster than that associated with AD, the most frequent neurodegenerative dementia. Another explanation is that an NABD accompanied by substantial axonal neurodegeneration and known to reveal long-term cognitive decline resembling that observed in AD patients should be distinguished from an autoimmune encephalopathy entity. CSF tau protein is known to be interrelated with cognitive functions in AD and other cognitive impairment phases (23), but so far, no study has investigated its correlation in NABD. As tau protein is elevated when axonal neurodegeneration occurs, it is not surprising that both the long-term and yearly decline of cognitive dysfunction is related to total tau protein. We thus detected mixed pathologies in most of our patients with neural autoantibodies, namely, 1) neural autoantibodies implying CNS inflammation and 2) elevated tau protein in 35.7% and ptau181 in 71.4% of patients as indicators of neurodegeneration. Our results show that total tau protein is a potentially useful biomarker for cognitive dysfunction in both AD and NABD patients. Furthermore, AD patients reveal a non-significant tendency towards higher tau-protein levels as a potential biomarker to differentiate between groups. Total tau protein thus seems to be a better marker of disease progression in NABD, as indicated by long-term cognitive decline, than ptau181. However, we should keep in mind that the axonal neurodegeneration indicated by the total tau protein is higher in AD than in NABD. As ptau181 is an established marker of disease progression in AD patients (24), we were surprised to find that it failed to correlate in our cohort suffering from longitudinal cognitive decline. The possible reasons for this might be our small sample and heterogeneous cohort involving early-onset and late-onset AD patients. Nevertheless, total tau protein concentrations in CSF correlated with the cognitive status reflected by the MMSE score in 4 of the 5 large datasets from the longitudinal ADNI studies (The Alzheimer’s Disease Neuroimaging Initiative), INDD (The Integrated Neurodegenerative Disease Database), and CBAS (The Czech Brain Aging Study) as well as the DESCRIPA cross-sectional study (Development of Screening Guidelines and Criteria for Predementia Alzheimer’s Disease) that were analyzed by the Global Alzheimer’s Association Interactive Network (GAAIN) database (25). However, in the cross-sectional EPINETTE dataset (database of patients consulting the university hospital HUG memory center in Geneva Switzerland), such a correlation between cognitive dysfunction and the CSF total tau concentration was not detected, as verified in the Eckhoff et al. (25) analysis. These differences might be associated with heterogeneous study designs and cohorts; most concur with our results in terms of cognitive dysfunction in association with total CSF tau protein levels. High CSF total tau levels were associated with faster loss of cognitive dysfunction in an AD cohort independent of their AD stage in a study by Duits et al. (26), but also in non-demented subjects—evidence suggests that different tau-level subgroups may reflect distinct underlying biological processes and that tau levels are not markers of disease progression per se. Our results indicate a non-significant but visible trend in **Figure 2** that, compared to our NABD patients, our AD patients had higher t-tau levels and higher MMSE score differences as measures of progressing cognitive decline, which warrants further investigation in a larger patient cohort. Higher total CSF tau levels in older subjects are associated with the hippocampal activity level and object discrimination (27), suggesting that tau pathology might affect hippocampal memory formation not only in AD patients but also in the elderly in general and in those with other diseases involving hippocampal pathology such as NABD. Körtvelyessy et al. (28) showed that the occurrence of high CSF tau was associated with signs of temporal lobe damage in MRI in cell-surface autoantibody-positive encephalitis, underpinning the phenomenon of axonal neurodegeneration in autoimmune cognitive impairment. However, no study to date has investigated total tau protein levels and cognitive dysfunction in neural autoantibody-associated cognitive decline, a cognitive decline that seems to mirror that observed in AD. The current definition of autoimmune dementia (3, 4) does not imply axonal neurodegeneration as a relevant diagnostic factor. However, if verifiable in larger patient cohorts, our findings might eventually prove that axonal neurodegeneration is in fact a relevant aspect of NABD that can be designated as autoimmune dementia. Our results are therefore highly relevant to arriving at a new definition of autoimmune dementia. These results suggest that the detection of neural autoantibodies might reflect an ongoing inflammatory state triggering axonal degeneration or one that is secondary to progressing neurodegeneration in patients who reveal no Alzheimer’s pathology in CSF. The latter hypothesis is supported by a recent study by Giannocoro et al. (29) that investigated the frequency of neural autoantibodies in the sera of 93 patients with neurodegenerative disease. Their working group detected autoantibodies targeting neural cell-surface antigens in 13.8% of patients with similar autoantibodies such as GlycinR and CASPR2. Furthermore, they identified an irregular disease course as one important predictor for detecting autoantibodies. These findings concur with the tendency towards relevantly less cognitive dysfunction, revealing its rapidly modulating nature, which could reflect an irregular disease course. Furthermore, recent animal studies demonstrated that GluA3 AMPAR autoantibodies can alter nervous system microstructures such as dendrites in mice (30), confirming our hypothesis that the association between autoantibodies and neurodegeneration in some of our NABD patients might be causally linked.

### Limitations

An important limitation of our study is the heterogeneous group of patients with NABD, as we specified different neural autoantibodies. Furthermore, we formed a control group of AD patients who were age- and gender-matched but who we did not differentiate as early- and late-onset AD patients often sharing a distinct neuropsychological profile entailing an either parietally or temporally predominant affection. However, as we detected no neuropsychological differences between groups, we need not subclassify AD patients further into those with an early onset versus those with a late AD onset. Cognitive decline is only assessed *via* the MMSE, not the entire CERAD testing procedure, which is a more global measurement that does not encompass all cognitive subdomains. Furthermore, we cannot rule out that more comprehensive cognitive markers are affected more strongly in NABD than AD. A further limitation is that we cannot exactly estimate the prevalence of NABD. A recent study investigated neural autoantibodies in 26 of 154 patients revealing possible clues for autoimmunity, as specific indicators were present (2). In that study (2), we detected neural autoantibodies associated with cognitive decline in 58% of our patients. No study to date has questioned NABD’s prevalence in a larger cohort simply because testing neural autoantibodies is so costly. It is currently limited to those patients presenting specific clinical features, suggesting a higher likelihood of a positive antibody result. Another caveat is that we clearly state that our study design does not aim to compare the prevalence of neural autoantibodies in dementia compared to patients with AD. In that case, we would have had to test for neural autoantibodies equally in both groups. However, our clear aim was to see if there is any clinical or laboratory profile or pattern distinguishing patients with neural autoantibodies from those with classical AD that might prove diagnostically relevant before expensive autoantibody testing is initiated. Our pilot data showed that a lower but often pathological level of total tau protein is a potential candidate for such a profile, which needs to be assessed in a larger independent patient cohort to be examined together with other groups suffering from neurodegenerative dementia. Although we cannot provide a strong link between the clinical dementia presentation and laboratory data, our research data point to a new research direction as a proof-of-concept study that warrants being reported and which also requires reproduction in larger independent patient cohorts.

### Conclusions

Taken together, our results deliver evidence of cognitive dysfunction that progresses similarly in NABD and AD patients, thereby implying that the progression of cognitive dysfunction long-term is no biomarker that distinguishes NABD from AD. However, the total tau protein in CSF might prove to be a relevant biomarker of a disease mechanism or of disease progression that should be investigated in large-scale studies and which might later be implemented to better characterize the NABD disease entity. Total tau protein levels are higher in AD than in NABD, thus suggesting the usefulness of tau protein as a biomarker before assessing neural autoantibodies in patients. The higher total tau levels in AD patients compared to NABD patients are probably not a result solely of the disease’s duration and ongoing neurodegeneration, as the time between disease onset and of assessing the CSF and neural autoantibodies and neurodegeneration markers did not differ between groups. It is tempting to postulate that if high tau levels are observed, the detection of neural autoantibodies is less likely to be associated with a positive autoantibody result. If t-tau protein is also confirmed in larger-scale studies to be higher in patients with AD than in those with autoantibody-associated cognitive decline, t-tau protein might serve as an additional diagnostic tool to differentiate between AD patients and those with anti-neural autoantibody-associated cognitive impairment. No clinical features seem to be important for differentiating these two groups before neural autoantibodies are assessed. This might be due to autoimmune indicators (20) mainly derived from strategies for selecting autoimmune encephalitis patients (31) and various autoantibody-associated psychiatric syndromes (20), but not for patients with autoantibody-associated dementia. Autoimmune indicators appear inadequate and should be tested in larger patient cohorts to see if they are sensitive and specific enough for detecting particular features of patients with NABD. However, focal dysfunction in the sensory or motor system and deficits in speaking abilities (aphasia, dysarthria, or mutism) in addition to dementia seem to be clinical features that occur in NABD patients, and that warrant further investigation in large-scale studies to validate their diagnostic significance in differentiating these dementia entities. Our pilot data show that NABD is a novel disease spectrum deserving more research so as to develop better diagnostic and treatment guidelines. Furthermore, NABD seems to be a hybrid between autoimmunity and neurodegeneration that might unveil highly relevant clues about autoimmune and neurodegenerative processes and their complex interaction—all of which would help us better understand this disease’s pathogenesis.

## Data Availability Statement

The raw data of the supporting conclusions of this article will be made available from the corresponding author on demand.

## Ethics Statement

The study involving human participants was reviewed and approved by the Ethical Committee of the University Medical Center Göttingen. Written informed consent for participation was not required for this study in accordance with the national legislation and the institutional requirements.

## Author Contributions

NH and CB wrote the manuscript. AJ, CB, IG, and SH contributed to the data collection. AJ, CB, BT, BM, CT, DF, and JW revised the manuscript for important intellectual content. All authors listed have made a substantial, direct, and intellectual contribution to the work and approved it for publication.

## Funding

Funding was obtained from the Open Access Fund of the University of Göttingen.

## Conflict of Interest

The authors declare that the research was conducted in the absence of any commercial or financial relationships that could be construed as a potential conflict of interest.

## Publisher’s Note

All claims expressed in this article are solely those of the authors and do not necessarily represent those of their affiliated organizations, or those of the publisher, the editors and the reviewers. Any product that may be evaluated in this article, or claim that may be made by its manufacturer, is not guaranteed or endorsed by the publisher.
